# Long-term psychosocial and socioeconomic outcomes after living kidney donation: a single-center Romanian cohort study

**DOI:** 10.25122/jml-2026-0052

**Published:** 2026-06

**Authors:** Lucas-Gabriel Discălicău, Cătălin Baston, Oana Moldoveanu, Alexandru Iordache, Teodor Căluşi, David Arnăutu, Ioanel Sinescu

**Affiliations:** 1 Carol Davila University of Medicine and Pharmacy, Bucharest, Romania; 2 Center of Surgical Urology and Kidney Transplantation, Fundeni Clinical Institute, Bucharest, Romania; 3 Anesthesiology and Intensive Care Unit, Fundeni Clinical Institute, Bucharest, Romania

**Keywords:** living kidney donation, donor outcomes, quality of life, psychosocial outcomes, financial burden, long-term follow-up, eGFR

## Abstract

Living kidney donation is the treatment of choice for patients with end-stage kidney disease, yet the long-term psychosocial and socioeconomic consequences for donors remain incompletely characterized, particularly in Eastern European cohorts. This study aimed to assess the long-term psychosocial and socioeconomic impact of living kidney donation in a single-center Romanian cohort and to compare donor-reported outcomes by kidney function at last follow-up. We conducted a retrospective cross-sectional assessment of living kidney donors who underwent nephrectomy at a single Romanian transplant center between January 2015 and December 2019. Of an initial cohort of 142 living kidney donors, six had died during follow-up; the remaining 136 donors were evaluated at a mean follow-up of 100.97 ± 17.89 months. Adverse outcomes were stratified by kidney function at last follow-up (eGFR < 45 vs ≥ 45 mL/min/1.73 m^2^). Overall, living kidney donors reported no regret (0%), no family disruption (0%), no financial burden (0%), and no occupational change (0%), along with low rates of depression (5.1%), anxiety (5.9%), and fear of kidney failure (2.2%). However, 10.3% reported they would not repeat donation if given the choice, and this unwillingness was markedly higher among donors who had reached eGFR < 45 mL/min/1.73 m^2^ at last follow-up (33.3% vs 6.8%, *P* = 0.003). Long-term psychosocial outcomes after living kidney donation are overall favorable, but long-term reduced kidney function was associated with donor reluctance to repeat donation, underscoring the importance of rigorous donor selection and careful monitoring of kidney function.

## Introduction

Kidney transplantation is the optimal treatment for end-stage kidney disease, conferring longer survival, better quality of life, and lower long-term costs compared with maintenance dialysis [1]. Living donor transplantation offers additional advantages over deceased-donor grafts, including shorter waiting time, the possibility of pre-emptive transplantation, and superior long-term graft survival [1,2]. In many countries, living donors now account for a substantial proportion of the kidney transplant activity, and current clinical guidelines endorse the careful expansion of donor selection criteria to address the persistent gap between demand and organ supply [2].

While the medical and surgical safety of living kidney donation has been extensively documented, the long-term psychosocial and socioeconomic consequences for donors are less studied. Most donors report health-related quality of life comparable to the general population, but a minority experience adverse outcomes including depression, anxiety, regret, and donation-related financial strain [3–5]. The Renal and Lung Living Donors Evaluation Study (RELIVE) and subsequent large cohorts have shown that longer recovery time, increased financial burden, and a sense of moral obligation are among the factors most consistently associated with post-donation distress [6,7]. Beyond mental health, concerns about future kidney failure, changes in body image following nephrectomy, and disruption of professional or family life are also well-recognized themes in the donor experience [8–10].

Despite this growing evidence base, data from Eastern European transplant programs, including Romania, remain scarce. Cultural, economic, and healthcare system differences may influence both the objective consequences of donation (particularly financial) and donors’ subjective evaluation of their decision. Moreover, few studies have systematically examined the relationship between long-term renal function and donor-reported psychosocial outcomes, even though donors who experience significant kidney function decline may be the group at highest risk for regret and distress [11]. With a mean follow-up exceeding eight years, our cohort offers an opportunity to address this gap.

This study aimed to assess the long-term psychosocial and socioeconomic impact of living kidney donation in a single-center Romanian cohort and to compare donor-reported outcomes between those who maintained preserved kidney function and those whose estimated glomerular filtration rate (eGFR) fell below 45 mL/min/1.73 m^2^ at last follow-up.

## Material and methods

### Study design and population

We performed a single-center, retrospective cross-sectional study of all living kidney donors who underwent donor nephrectomy at the Center of Surgical Urology and Kidney Transplantation, Fundeni Clinical Institute, Bucharest, Romania, between January 2015 and December 2019. Of the initial 142 donors, six had died during follow-up from causes unrelated to donation and were excluded from the analysis. The final study cohort comprised 136 donors. The mean follow-up period was 100.97 ± 17.89 months. All donors were evaluated and selected in accordance with our institutional protocol, which is aligned with the Kidney Disease: Improving Global Outcomes (KDIGO) Clinical Practice Guideline on the Evaluation and Care of Living Kidney Donors [2].

### Data collection and variables

Baseline donor characteristics collected at the time of donation included age, sex, donor-recipient relationship, body mass index (BMI) and obesity status, smoking status, pre-donation hypertension and dyslipidemia, number of comorbidities, serum creatinine, and estimated glomerular filtration rate (eGFR). Post-donation variables recorded at last follow-up included de novo hypertension, de novo dyslipidemia, and de novo type 2 diabetes mellitus. Donor-reported psychosocial and socioeconomic outcomes were assessed through a structured questionnaire administered during follow-up visits or by telephone interview, and covered twelve domains: (i) perceived impact on social life; (ii) perceived impact on professional life; (iii) negative impact on family life; (iv) regret regarding the decision to donate; (v) willingness to donate again if they could; (vi) perceived change in body image; (vii) symptoms of depression; (viii) symptoms of anxiety; (ix) perceived stress attributable to donation; (x) fear of future kidney failure; (xi) donation-related financial burden; and (xii) change in occupation attributable to donation.

### Outcome stratification

Donors were stratified according to kidney function at last follow-up, using an eGFR threshold of 45 mL/min/1.73 m^2^ (CKD stage 3b) to define the two comparison groups (eGFR ≥ 45 vs eGFR < 45 mL/min/1.73 m^2^). This threshold was chosen because it represents a clinically meaningful level of kidney function loss, beyond which cardiovascular and renal outcomes are known to diverge [2].

### Statistical analysis

Continuous variables are presented as mean ± standard deviation (SD), and categorical variables as counts and percentages. Differences in categorical outcomes between the two eGFR strata were analyzed using the Fisher exact test. A two-sided *P* value < 0.05 was considered statistically significant. All analyses were performed using SPSS version 26. The study was approved by the Institutional Review Board of Fundeni Clinical Institute, and all donors provided written informed consent for the use of their clinical and follow-up data.

## Results

### Baseline characteristics of the cohort

Baseline and follow-up characteristics of the 136 living kidney donors are summarized in Table 1. Mean age at donation was 52.15 ± 11.29 years, and 101 donors (74.3%) were female. The vast majority of donations were from first-degree relatives (130, 95.6%), with second- and third-degree relationships accounting for 2.9% and 1.5%, respectively. At the time of donation, 36 donors (26.5%) had hypertension, 44 (32.4%) had dyslipidemia, 15 (11.0%) were obese, and 27 (19.9%) were active smokers; 38 donors (27.9%) had a single comorbidity, and 14 (10.3%) had two or more. Mean pre-donation serum creatinine was 0.86 ± 0.18 mg/dL, and mean eGFR was 88.79 ± 18.74 mL/min/1.73 m^2^. At last follow-up, 19 donors (14.0%) had developed de novo hypertension, 24 (17.6%) de novo dyslipidemia, and 2 (1.5%) de novo type 2 diabetes mellitus (Table 1).

**Table 1:** Baseline characteristics of the 136 living kidney donors at donation and at last follow-up

Characteristic	Donors (*n* = 136)
**Characteristics at donation**	
Age, years (mean ± SD)	52.15 ± 11.29
Female sex, *n* (%)	101 (74.3)
Donor–recipient relationship, *n* (%)	
First-degree relative	130 (95.6)
Second-degree relative	4 (2.9)
Third-degree relative	2 (1.5)
Hypertension, *n* (%)	36 (26.5)
Dyslipidemia, *n* (%)	44 (32.4)
Obesity, *n* (%)	15 (11.0)
Active smoking, *n* (%)	27 (19.9)
1 comorbidity, *n* (%)	38 (27.9)
≥2 comorbidities, *n* (%)	14 (10.3)
Serum creatinine, mg/dL (mean ± SD)	0.86 ± 0.18
eGFR, mL/min/1.73 m^2^ (mean ± SD)	88.79 ± 18.74
**Characteristics at last follow-up**	
De novo hypertension, *n* (%)	19 (14.0)
De novo dyslipidemia, *n* (%)	24 (17.6)
De novo type 2 diabetes mellitus, *n* (%)	2 (1.5)

### Psychosocial and socioeconomic outcomes

Donor-reported outcomes across the twelve psychosocial and socioeconomic domains are presented in Table 2. Overall, adverse outcomes were uncommon. No donor reported regret regarding the decision to donate, no donor reported a negative impact on family life, no donor reported a donation-related financial burden, and no donor reported a change in occupation attributable to donation. Perceived impact on social life and on professional life were each reported by two donors (1.5%), and two donors (1.5%) reported a change in body image. Symptoms of depression were reported by seven donors (5.1%), anxiety by eight donors (5.9%), stress by seven donors (5.1%), and fear of future kidney failure by three donors (2.2%). However, 14 donors (10.3%) stated they would not repeat the donation if given the choice, despite no explicit regret.

**Table 2:** Donor-reported psychosocial and socioeconomic outcomes at last follow-up, stratified by eGFR

Outcome	Entire cohort (*n* = 136)	eGFR ≥ 45 mL/min at last FU (*n* = 118)	eGFR < 45 mL/min at last FU (*n* = 18)	*P* value
Impact on social life	2 (1.5)	1 (0.8)	1 (5.6)	0.24
Impact on professional life	2 (1.5)	2 (1.7)	0 (0)	0.75
Negative impact on family life	0 (0)	0 (0)	0 (0)	1.00
Regrets donation	0 (0)	0 (0)	0 (0)	1.00
**Would not repeat donation**	**14 (10.3)**	**8 (6.8)**	**6 (33.3)**	**0.003**
Change in body image	2 (1.5)	2 (1.7)	0 (0)	1.00
Depression	7 (5.1)	7 (5.9)	0 (0)	0.59
Anxiety	8 (5.9)	8 (6.8)	0 (0)	0.59
Stress	7 (5.1)	7 (5.9)	0 (0)	0.59
Fear of kidney failure	3 (2.2)	3 (2.5)	0 (0)	1.00
Financial burden	0 (0)	0 (0)	0 (0)	1.00
Change in occupation	0 (0)	0 (0)	0 (0)	1.00

Values are *n* (%). *P* values from Fisher's exact test. FU, follow-up; eGFR, estimated glomerular filtration rate.

### Outcomes stratified by kidney function at last follow-up

When stratified by eGFR at last follow-up, 118 donors (86.8%) maintained an eGFR ≥ 45 mL/min/1.73 m^2^ while 18 donors (13.2%) had reached an eGFR < 45 mL/min/1.73 m^2^ (Table 2). The only outcome with a statistically significant difference between the two strata was willingness to donate again. Donors with eGFR < 45 mL/min/1.73 m^2^ were markedly less likely to report they would repeat donation than those with preserved function (33% vs 6.8%, *P* = 0.003; Table 2). Symptoms of depression (5.9%), anxiety (6.8%), stress (5.9%), and fear of kidney failure (2.5%) were exclusively observed in the preserved-function group, but these differences did not reach statistical significance. No differences were observed for the remaining outcomes.

## Discussion

This single-center study provides the longest follow-up assessments of psychosocial and socioeconomic outcomes in living kidney donors published to date, with a mean follow-up approaching 9 years. Our findings confirm the overall favorable long-term profile of living kidney donation and, at the same time, identify a clinically meaningful signal: donors who had progressed to an eGFR below 45 mL/min/1.73 m^2^ were five times more likely to state that they would not repeat the decision to donate, despite not reporting explicit regret. This observation carries direct implications for donor selection and long-term follow-up.

Only two donors (1.5%) in our cohort reported a perceived negative impact on social life, a finding consistent with the broader literature. Systematic reviews of long-term donor outcomes have shown that social functioning remains unchanged or improves after donation in most donors, and a recent review emphasized that social connection and perceived social support are central emotional needs in the post-donation period [12,13]. Our very low rate may partly reflect cultural factors in a cohort dominated by first-degree relatives, where donation is often framed as a family act rather than an individual sacrifice.

Two donors (1.5%) reported a perceived impact on professional life. Published evidence indicates that most donors return to work within four to 6 weeks after nephrectomy, strongly influenced by the physical demands of their occupation [14,15]. In a U.S. cohort of 629 donors, longer time to return to work was a significant predictor of donation-related financial burden, and donors in manual or skilled-trade occupations experienced the greatest burden per week away from work [15]. The low rate of reported occupational impact in our cohort is consistent with an older donor population (mean age 52 years) who are predominantly in non-manual employment, but may also reflect under-reporting in a cultural context where donation is viewed positively.

No donor in our cohort reported a negative impact on family life. In a systematic review of 51 studies encompassing more than 5,000 donors, 83–100% reported no change or an improved relationship with family members, spouses, and non-recipient children after donation [16]. Qualitative work further shows that donation often strengthens interpersonal ties, although renegotiation of family roles and occasional emotional lability have been described [17]. Our result is in line with this overall favorable picture, particularly given the high proportion of first-degree donations in our cohort.

No donor in our cohort expressed explicit regret regarding the decision to donate. However, 14 donors (10.3%) stated they would not repeat donation if they could, a proportion slightly higher than the 0–7% range reported in contemporary systematic reviews [12,18]. Our data show that the willingness to repeat donation is strongly dependent on subsequent renal function: among donors with eGFR < 45 mL/min/1.73 m^2^ at last follow-up, one-third (33.3%) stated they would not donate again, compared with only 6.8% of donors with preserved function (*P* = 0.003). This finding is novel and clinically important. Even in the absence of conscious regret, an eGFR threshold traditionally considered the boundary between mild and moderate chronic kidney disease appears to function as a cognitive landmark that reshapes donors’ retrospective evaluation of their decision. It directly reinforces the importance of rigorous donor selection criteria, particularly careful assessment of cardiometabolic risk factors such as hypertension, obesity, smoking, and prediabetes, and the need for lifelong renal surveillance, consistent with current KDIGO guidance [2].

We observed symptoms of depression in 5.1%, anxiety in 5.9%, and stress in 5.1% of donors. These rates are closely aligned with those reported in large Western cohorts: 5.5% anxiety and 4.2% depression in a screening study of 825 donors using the Generalized Anxiety Disorder-2 (GAD-2) and Patient Health Questionnaire-2 (PHQ-2) screening tools [19], and 8% depressive symptoms in the RELIVE cohort using the Patient Health Questionnaire-9 (PHQ-9). Factors such as longer recovery time, greater financial burden, feelings of moral obligation, younger age, and lower physical quality of life were identified as contributors to post-donation depressive symptoms [6].

A recent systematic review of living organ donors similarly found that post-donation rates of depression and anxiety are largely comparable to those of the general population. However, a minority of donors experience clinically significant distress [3].

Only three donors (2.2%) reported fear of future kidney failure. The Fear of Kidney Failure questionnaire, developed and validated in a cohort of 364 former donors, reported that 13% of donors experienced moderate-to-high fear of future renal failure, with non-White race, genetic relationship with the recipient, and low satisfaction with the donation experience emerging as independent predictors [10]. The absolute lifetime risk of end-stage renal disease after donation, though higher than in matched healthy non-donors, remains low — approximately 3.5 per 1,000 over 15 years in the landmark study of Muzaale and colleagues [11]. Our low rate of donation-related renal anxiety likely reflects sustained reassurance from structured follow-up, a protective mechanism previously emphasized in the literature [10,16].

The central clinical message of our study is the strong association between post-donation eGFR < 45 mL/min/1.73 m^2^ and the donor’s stated unwillingness to repeat donation if they could. This finding supports current international guidance emphasizing rigorous pre-donation risk stratification, particularly the identification of donors at elevated risk of long-term loss of kidney function [2]. Pre-donation factors known to predict post-donation eGFR trajectory include age, pre-donation eGFR, BMI, hypertension, prediabetes, and smoking [2,20]. Our data suggest that the psychological cost of kidney function decline is not trivial and may extend beyond the traditional medical endpoints, arguing for (i) structured use of validated risk-prediction tools in the selection process, (ii) transparent pre-donation counseling regarding long-term renal outcomes, and (iii) lifelong renal monitoring with early intervention for modifiable risk factors.

The main strengths of this study are the long mean follow-up of more than 8 years, the near-complete capture of a consecutive single-center cohort, and the simultaneous assessment of 12 psychosocial and socioeconomic domains, with stratification by long-term renal function. Several limitations must be acknowledged. First, the cross-sectional design does not allow assessment of change from a pre-donation baseline, and reliance on donor self-report introduces recall and social desirability biases, which have been specifically highlighted as confounders in donor psychosocial research [13,18]. Second, the questionnaire used in this study was center-developed rather than derived from validated, widely used instruments such as the Short Form-36 (SF-36), PHQ-9, GAD-7, or the Fear of Kidney Failure scale, thereby limiting direct comparability with international data. Third, the eGFR < 45 mL/min/1.73 m^2^ subgroup is small (*n* = 18), reducing statistical power for secondary outcomes. Fourth, the financial burden in our cohort may be underestimated because indirect costs may have been overlooked. Prospective, multicenter studies using validated instruments are needed to confirm these findings in the Eastern European context.

## Conclusion

In this Romanian cohort of living kidney donors with long-term follow-up, psychosocial and socioeconomic outcomes were overall favorable. We found an association between decreased long-term kidney function and unwillingness to repeat donation. These findings reinforce the importance of stringent donor selection criteria, structured pre-donation counseling about long-term renal risk, and lifelong follow-up to preserve both the kidney health and the long-term psychological well-being of living kidney donors.

## Data Availability

The data that support the findings of this study are available from the corresponding author upon reasonable request.

## References

[1] Wolfe RA, Ashby VB, Milford EL, Ojo AO, Ettenger RE, Agodoa LY (1999). Comparison of mortality in all patients on dialysis, patients on dialysis awaiting transplantation, and recipients of a first cadaveric transplant. N Engl J Med.

[2] Lentine KL, Kasiske BL, Levey AS, Adams PL, Alberú J, Bakr MA (2017). KDIGO Clinical Practice Guideline on the Evaluation and Care of Living Kidney Donors. Transplantation.

[3] Ong QL, Lim LJH, Ho RCM, Ho CSH (2021). Depression, anxiety, and associated psychological outcomes in living organ transplant donors: a systematic review. Gen Hosp Psychiatry.

[4] Massey EK, Rule AD, Matas AJ (2025). Living Kidney Donation: A Narrative Review of Mid-and Long-term Psychosocial Outcomes. Transplantation.

[5] Clemens KK, Thiessen-Philbrook H, Parikh CR, Yang RC, Karley ML, Boudville N (2006). Donor Nephrectomy Outcomes Research (DONOR) Network. Psychosocial health of living kidney donors: a systematic review. Am J Transplant.

[6] Jowsey SG, Jacobs C, Gross CR, Hong BA, Messersmith EE, Gillespie BW (2014). RELIVE Study Group. Emotional well-being of living kidney donors: findings from the RELIVE Study. Am J Transplant.

[7] Messersmith EE, Gross CR, Beil CA, Gillespie BW, Jacobs C, Taler SJ (2014). RELIVE Study Group. Satisfaction With Life Among Living Kidney Donors: A RELIVE Study of Long-Term Donor Outcomes. Transplantation.

[8] Johnson EM, Anderson JK, Jacobs C, Suh G, Humar A, Suhr BD (1999). Long-term follow-up of living kidney donors: quality of life after donation. Transplantation.

[9] Menjivar A, Torres X, Manyalich M, Fehrman-Ekholm I, Papachristou C, de Sousa-Amorim E (2020). Psychosocial risk factors for impaired health-related quality of life in living kidney donors: results from the ELIPSY prospective study. Sci Rep.

[10] Rodrigue JR, Fleishman A, Vishnevsky T, Whiting J, Vella JP, Garrison K (2014). Development and validation of a questionnaire to assess fear of kidney failure following living donation. Transpl Int.

[11] Muzaale AD, Massie AB, Wang MC, Montgomery RA, McBride MA, Wainright JL (2014). Risk of end-stage renal disease following live kidney donation. JAMA.

[12] Cazauvieilh V, Moal V, Prudhomme T, Pecoraro A, Piana A, Campi R (2023). Psychological Impact of Living Kidney Donation: A Systematic Review by the EAU-YAU Kidney Transplant Working Group. Transpl Int.

[13] Colonnello V, La Manna G, Cangini G, Russo PM (2024). Post-Donation Evaluation: Emotional Needs for Social Connection and Social Support among Living Kidney Donors-A Systematic Review. Healthcare (Basel).

[14] Przech S, Garg AX, Arnold JB, Barnieh L, Cuerden MS, Dipchand C (2018). Donor Nephrectomy Outcomes Research (DONOR) Network. Financial Costs Incurred by Living Kidney Donors: A Prospective Cohort Study. J Am Soc Nephrol.

[15] Larson DB, Wiseman JF, Vock DM, Berglund DM, Roman AM, Ibrahim HN (2019). Financial burden associated with time to return to work after living kidney donation. Am J Transplant.

[16] Clemens K, Boudville N, Dew MA, Geddes C, Gill JS, Jassal V (2011). Donor Nephrectomy Outcomes Research (DONOR) Network. The long-term quality of life of living kidney donors: a multicenter cohort study. Am J Transplant.

[17] Ralph AF, Butow P, Craig JC, Wong G, Chadban SJ, Luxton G (2019). Living kidney donor and recipient perspectives on their relationship: longitudinal semi-structured interviews. BMJ Open.

[18] Timmerman L, Zuidema WC, Erdman RA, Kranenburg LW, Timman R, Ijzermans JN (2013). Psychologic functioning of unspecified anonymous living kidney donors before and after donation. Transplantation.

